# Combined debridement, bone graft and articular cavity sealing using synovium in treating metaphyseal osteomyelitis involving knee joints

**DOI:** 10.3892/etm.2012.762

**Published:** 2012-10-25

**Authors:** WEIJU LU, GANG LIU, BIN LI, NINGWEN SHI, JIANNING ZHAO

**Affiliations:** Department of Orthopedic Surgery, Jinling Hospital, Nanjing University School of Medicine, Nanjing, Jiangsu 210002, P.R. China

**Keywords:** osteomyelitis, knee joint, synovium sealing, bone graft

## Abstract

The aim of this study was to investigate the efficacy of combined debridement, bone graft and articular cavity sealing using synovium in the treatment of metaphyseal osteomyelitis involving the knee joint. Eleven patients with metaphyseal osteomyelitis, which involved femurs in 4 patients and tibiae in 7, were included. The patients received a novel treatment, which combined debridement, bone graft and articular cavity sealing using the synovium. Of the 11 patients, 4 patients with knee joint instability received a structural allograft and 7 with a stable knee joint underwent a particulate bone graft. The 11 patients underwent regular clinical and radiological evaluation; the average follow-up was 74 months (range, 58–96). Infection recurrence in the joint and bone graft area was not observed in 10 of the 11 cases. In one patient, who underwent a lateral granular cancellous bone allograft in the right tibial plateau, the infection recurred 2 weeks later in the graft area. The infection was arrested 3 months after re-debridement and a bilateral ilium bone graft to eliminate the dead space. Combined debridement, bone graft and articular cavity sealing using the synovium may be a feasible treatment for metaphyseal osteomyelitis involving the knee joint.

## Introduction

The distal femur and proximal tibia which constitute the knee joint are mainly cancellous bone covered by cartilage. Once comminuted fracture occurs, compression bone defects and massive broken bones often follow, usually involving the cartilage surface. Therefore, post-traumatic osteomyelitis at this region may easily cause cancellous bone and cartilage surface infection and necrosis. Purulent fluid flow into the articular cavity through fracture gaps, which may easily cause bone and joint infection, and the treatment remains difficult. The current study describes our experience of 11 cases of metaphyseal osteomyelitis, which involved 4 femurs and 7 tibiae, treated with debridement, bone graft and sealing of the articular cavity using the synovium.

## Patients and methods

### Patient data collection

From 1999 to 2003, 11 patients with metaphyseal osteomyelitis involving the knee joint were treated in our hospital. Ten of the patients were male and one was female, with a mean age of 38 years (range, 27–56). Four cases involved the distal femur (3 cases at the lateral condyle and 1 at the medial condyle), of which 2 cases resulted in bone nonunions. Seven cases involved proximal tibiae (4 cases at the lateral plateau and 3 at the medial plateau) and the bones healed fully. Three of the 11 cases remained with an implant (1 case with a retrograde locking nail in the femur and 2 with a lateral plate in the tibia) and an average of 2 surgical procedures (range, 1–4) had been undertaken by patients prior to administration (Table I).

After the patients were admitted, X-ray radiography, contrast fistulography, MRI, CT and emission computed tomography (ECT) examination were performed to assess the extent of the foci; the size and position of the dead bone and the strength and stability of the involved bone were also assessed. Bacteriological tests of pus and tissues from the foci were examined routinely (Table I).

### Procedures

According to the lesion sites, a medial or lateral incision was made, extending 12 cm proximal or distal to the knee joint. After internal fixation appliances were removed, necrotic granulation, fibrous cicatrix tissue and sequestra in the involved bone cavity were cleared thoroughly, and the sclerotic bone was also mechanically removed, followed by repeated compressive flushing of the focus and articular cavity with isotonic saline. Following debridement, the knee joint lateral stability was evaluated as follows: if the residual bone was able to resist varus or valgus stress and there was no obvious lateral movement in the joint, the joint was classified as stable (2 cases involving the lateral femoral condyle and 5 cases involving the tibia, of which 3 affected the medial plateau and 2 affected the lateral plateau). Otherwise, the joint was classified as unstable (2 cases involving the distal femur, of which 1 affected the lateral condyle and the other the medial condyle, and 2 cases involving the lateral tibial plateau).

### Management of the bone defect and articular cavity following debridement

**Figure 1:**
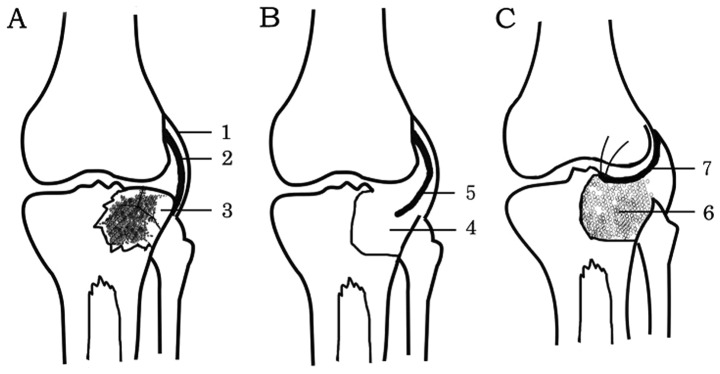
Schematic drawing of the surgical technique. (A) 1, Lateral capsular ligament; 2, thickened synovium; 3, osteomyelitis. (B) 4, Bone cavity after debridement; 5, dissociated thickened synovium. (C) 6, Granular cancellous bone graft; 7, sealing the joint cavity by covering the bone graft with the synovium.

**Figure 2:**
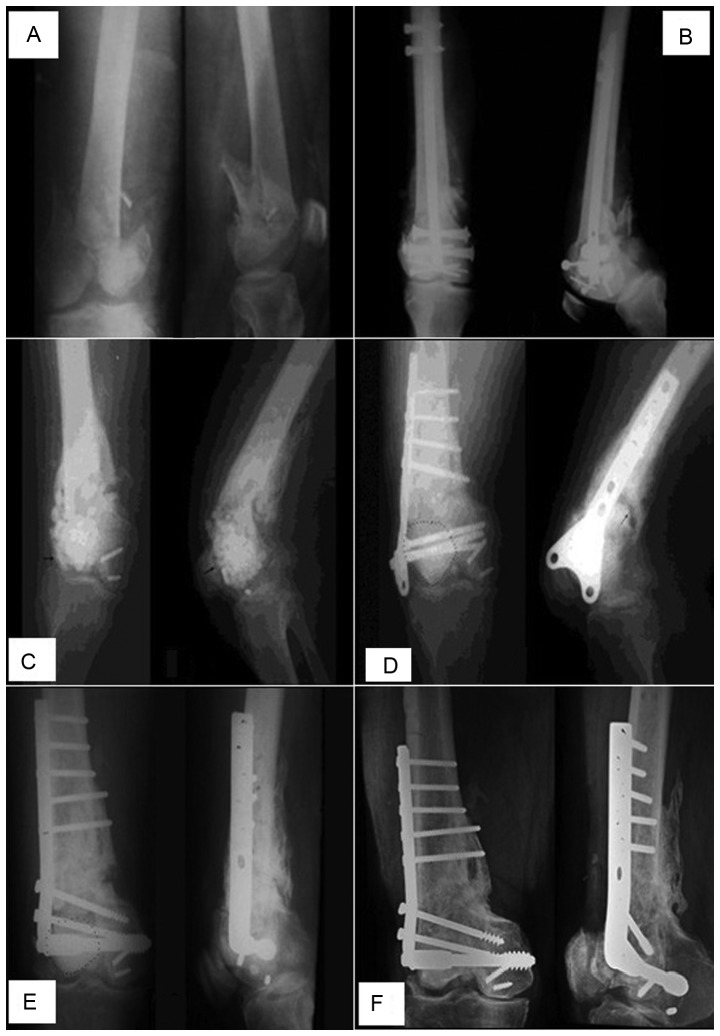
Postoperative infection of femoral condyle fracture. (A) Preoperative radiograph; (B) postoperative radiograph of internal fixation; (C) implantation of a vancomycin cement bead in the bone defect area (arrow) following debridement and dismantling of the internal fixation appliance; (D) implantation of a femoral condyle allograft from which the cartilage surface was removed (dotted line area) after the vancomycin cement bead had been removed and fixed by a strut plate; 8 months later, nonunion and displacement of the femoral condyle was observed (arrow indicates fracture line); (E) granulated cancellous autografting and a dynamic condylar screw plate was used to strengthen the fixation; this X-ray was produced 5 months after surgery (dotted line area indicates allograft); (F) 2 years after surgery, bone healing and existence of joint space was observed in the radiograph.

The following techniques were used after debridement: i) Single-stage bone graft and articular cavity sealing with synovium: of the 7 cases with stable knees, 4 cases (3 tibial and 1 femural) underwent a bilateral iliac granular cancellous autograft and the other 3 cases (2 tibial and 1 femoral), due to bone defects >40 ml, underwent a granular cancellous allograft. Of the 4 patients with unstable knees, 3 received primary bulk cancellous allografts (50, 65 and 110 ml) from which the cartilage surface had been removed prior to grafting, and cancellous allograft particles were implanted in order to bridge the gap between the host bone and allograft, and were fixed by a strut plate. The adjacent synovium was dissociated and sutured to seal the synovial articular cavity (Fig. 1). ii) Two-stage bulk allograft: 1 patient retained a femoral retrograde interlocking intramedullary nail and had an infection which affected the whole lateral condyle and resulted in the formation of a sequestrum. Infection spread to the distal medullary cavity and medial condyle along the locking nail. After debridement, the synovium was sutured to seal the articular cavity and a vancomycin cement bead was placed in the bone defect area. After 8 weeks, the vancomycin cement bead was removed, then, a femoral condyle allograft from which the cartilage surface had been removed was implanted and fixed using a strut plate. However, 8 months later, nonunion and displacement of the femoral condyle was observed in an X-ray, and a dynamic condylar screw plate was used to strengthen the fixation (Fig. 2). iii) Negative pressure drainage was used at the articular cavity and bone graft area.

### External fixation

Of the 11 cases, 10 underwent fixation for 2 months, 7 with a lateral and medial cast and 3 with a plastic brace. One patient who received a femoral medial condyle bulk bone graft underwent fixation by an external fixator with articulation for 6 months due to the uncertainty of plate fixation.

The removal time of the drainage tube was determined by the final volume of drainage (<5 ml/24 h). The average drainage times were 8 days for the articular cavity and 16 days for the bone graft area.

### Medication with antibiotics

Appropriate antibiotics were primarily selected according to the preoperative bacterial culture result and subsequently selected according to the culture result of the tissue obtained during surgery. Antibiotics were administered intravenously for 2–3 weeks from the day of surgery and then orally for 4 weeks. The patients underwent regular clinical and radiological evaluation. The average follow-up period was 74 months (range, 58–96).

## Results

Infection recurrence was not observed for 10 of the 11 cases in the joint and bone graft area. In one patient (no. 7 in Table I), who underwent a lateral granular cancellous bone allograft in the right tibial plateau, infection recurred 2 weeks later in the graft area. The patient underwent debridement and a bilateral ilium bone graft to eliminate the dead space, and 3 months later, the infection was arrested.

Of the 2 bone nonunion patients, bone union was achieved in 1 case while the other case (no. 1, in Table I) presented bone nonunion at 11 months after surgery. In this case, a revision surgery was performed to replace the implant with a dynamic condylar screw plate and an iliac autograft, and bone union was observed at the final follow-up. Slight bone reabsorption was observed in the granular cancellous bone autograft or allograft in the articular cavity surface, with no sign of bone loss in bulk allograft patients.

Two of the 11 (18%) cases developed a frontal misalignment of 15°, but were able to walk without crutches. Lateral instability of the knee joint was reported in 3 cases, of which 2 cases walked freely and 1 with crutches. The range of motion in all cases was >90°. No leg length discrepancy (>2 cm) was observed in the current study (Table I).

## Discussion

In metaphyseal osteomyelitis involving the knee joint, the primary infection usually originates from bone tissue. Therefore dead bone and the attached cartilage should be thoroughly removed during debridement, to eliminate the source of infection (1,2). Precise bone debridement is most effectively carried out with high speed burs and is performed until the ‘paprika sign’ (defined as scattered pinpoint bleeding indicating a good vasculature) is encountered (3).

Since the joint infection is secondary to the bone infection, after the granulation tissue on the cartilage surface and the synovial membrane had been removed, the articular cavity is able to resume normal metabolism and immune function. With the aid of effective antibiotics, the small quantity of bacteria remaining following debridement may be eliminated (4). No joint infection recurred in the 11 patients in this study, which demonstrated that the management option was reasonable and effective.

Bone defects resulting from debridement were reconstructed using granular cancellous bone grafts instead of other techniques, including vascularized bone grafting, distraction osteogenesis, bone graft substitutes and prosthetic devices (5–8).

Granular cancellous bone grafts have the following advantages: i) they are easily molded to fill any shape of bone defect, ii) the blood vessels of the defect cavity wall easily permeate the bone graft due to the abundant bone intergranular lattice channels, which may result in higher incorporation and union rates and, consequently, superior results (9,10). iii) Antibiotics and immuno-active substances exuded from the blood vessels are able to distribute well into the bone grafts, resulting in a strong ability to resist infection (11). Granular cancellous bone grafts also have disadvantages, for example, particulate bone grafts clearly have poorer mechanical properties than structural allografts (12). In 7 of the patients in the current study, the residual bone after debridement had the ability to resist lateral stress and therefore the joint was stable, with no need for stabilizing bone grafts.

Large osseous defects require bone for stability and are usually managed with a vascularized bone flap (13,14). The 4 patients in our study with large bone defects had joint instability; structural allografts from which cartilage had been removed beforehand and strut plate fixation were chosen in order to withstand a certain pressure, and stability of the joints was achieved.

The exposure of necrotic cartilage to the articular cavity is likely to increase the chances of infection recurring. The exposure of the bone graft to the articular cavity easily permits the bacteria remaining in the bone tissue to enter the articular cavity, or the inflammatory joint fluid to infiltrate the bone graft, both of which are counter to infection control. In addition, grafted bone extensively exposed to the articular cavity is not easily vascularized, requires a long time to be able to resist infection, may easily become a new source of infection and is likely to accelerate joint wear and degeneration.

Sealing the articular cavity with the highly vascularized synovium separates the articular cavity from the outside structures. The grafted bone may easily obtain a blood supply from the synovial membrane, which shortens the time taken to achieve revascularization (15).

In conclusion, the results of the 11 patients demonstrated that bone grafting combined with the sealing of the articular cavity using the synovium is an effective treatment option for metaphyseal osteomyelitis involving the knee joint. However, further study should be performed to investigate the long-term outcomes of the synovial tissue and the reconstructed joint using structural cortical allografts covered by the synovial membrane.

## Figures and Tables

**Table I:** Demographic and clinical characteristics of the patients.

Case	Gender	Age (years)	Infection site	Time interval (months)^a^	No. of surgeries before administration	Follow-up Bacterium species	(months)	Status of the knee (2 years following surgery)
1	M	56	LCF	9	3	MRSA	82	Lateral instability, walks with crutch, ROM: 0–90°
2	M	47	LTP	12	2	*Pseudomonas aeruginosa*	96	Normal
3	M	27	LCF	7	2	*Staphylococcus aureus*	74	Normal
4	M	33	MCF	6	1	*Staphylococcus aureus*	80	Lateral instability, walks freely, ROM: 0–100°
5	M	35	LCF	2	1	MRSA	90	Stable, ROM: 0–90°
6	M	36	MTP	5	2	*Staphylococcus aureus*	64	Stable, ROM: 0–130°
7	M	29	LTP	7	4	*Bacillus cloacae, Pseudomonas aeruginosa, Serratia marcescens*	73	Normal
8	F	42	MTP	12	1	*Staphylococcus aureus*	70	Normal
9	M	34	MTP	3	1	*Staphylococcus aureus*	67	Normal
10	M	39	LTP	9	2	MRSA	61	Stable, valgus 15°, ROM: 0–90°
11	M	43	LTP	11	2	*Bacillus cloacae*	58	Valgus 15°, walks freely, ROM: 0–100°

a Time interval since the previous surgery. M, male; F, female; MRSA, methicillin-resistant *Staphylococcus aureus*; LCF, lateral condyle of femur; MCF, medial condyle of femur; LTP, lateral tibial plateau; MTP, medial tibial plateau; ROM, range of motion.
